# The putative Cationic Amino Acid Transporter 9 is targeted to vesicles and may be involved in plant amino acid homeostasis

**DOI:** 10.3389/fpls.2015.00212

**Published:** 2015-04-01

**Authors:** Huaiyu Yang, York-Dieter Stierhof, Uwe Ludewig

**Affiliations:** ^1^Nutritional Crop Physiology, Institute of Crop Science, University of HohenheimStuttgart, Germany; ^2^Zentrum für Molekularbiologie der Pflanzen, University of TübingenTübingen, Germany

**Keywords:** amino acid permease, cationic amino acid, prevacuole, *trans*-Golgi network, nitrogen

## Abstract

Amino acids are major primary metabolites. Their uptake, translocation, compartmentation, and re-mobilization require a diverse set of cellular transporters. Here, the broadly expressed gene product of *CATIONIC AMINO ACID TRANSPORTER 9* (*CAT9*) was identified as mainly localized to vesicular membranes that are involved in vacuolar trafficking, including those of the *trans*-Golgi network. In order to probe whether and how these compartments are involved in amino acid homeostasis, a *loss-of-function cat9-1* mutant and ectopic over-expressor plants were isolated. Under restricted nitrogen supply in soil, *cat9-1* showed a chlorotic phenotype, which was reversed in the over-expressors. The total soluble amino acid pools were affected in the mutants, but this was only significant under poor nitrogen supply. Upon nitrogen starvation, the soluble amino acid leaf pools were lower in the over-expressor, compared with *cat9-1.* Over-expression generally affected total soluble amino acid concentrations, slightly delayed development, and finally improved the survival upon severe nitrogen starvation. The results potentially identify a novel function of vesicular amino acid transport mediated by *CAT9* in the cellular nitrogen-dependent amino acid homeostasis.

## Introduction

Plant roots are capable to efficiently take up mineral nitrogen in the form of ammonium and nitrate from soils, which are both transiently stored or immediately assimilated into amino acids. In addition to the uptake of nitrogen, a major focus to increase nitrogen use efficiency of crops targets the assimilation and remobilization of nitrogen during senescence and grain filling (Hortensteiner and Feller, 2002; Chardon et al., 2012; Pratelli and Pilot, 2014). The first primary product of ammonium assimilation, glutamine, its precursor glutamate, and the further products asparagine, aspartate, and serine often form the major pool of soluble amino acids in the leaves of many species. Depending on the plant species, these and sometimes a few other amino acids form the major long distance transport nitrogen metabolites, are precursors of various downstream metabolites, are required for protein biosynthesis and serve as nitrogen storage compounds (Xu et al., 2012; Pratelli and Pilot, 2014). The transient storage capacity of nitrate in vacuoles is significant and may reach up to 250 mM and is lower for ammonium, due to its toxicity at higher mM concentrations. The amino acid concentrations in vacuoles are typically surprisingly low, even under optimal N supply

(Riens et al., 1991). Amino acids may be transiently incorporated into peptide and proteins, which are degraded on demand. While soluble proteins are effectively degraded via the 26S proteasome, membrane protein degradation requires a membrane-delimited pathway, which includes the lytic vacuole. After degradation, the export of amino acids or peptides from the vacuolar lumen to the cytoplasm likely involves specific carriers, but the molecular mechanisms of amino acid transport across intracellular membranes are still relatively poorly understood. Although it is clear that amino acids can be transiently stored in the large central leaf vacuoles (Lohaus and Heldt, 1997), most amino acids are effectively excluded from the vacuolar lumen of photosynthetically active barley and spinach leaves (Winter et al., 1994). Only because of the large volume of the vacuolar lumen, the total amino acid content (but not the concentration) in vacuoles often exceeds that of the cytosol (De, 2000). The lytic vacuole, which is involved in the degradation of proteins appears to be derived from the endoplasmic reticulum and requires the export of amino acids from the lumen to support the metabolism in the cytosol. Loading and unloading of vacuolar amino acid content may be additionally mediated by dynamic vesicular transport, which also delivers proteins for degradation or storage to the vacuole.

The molecular identity of transporters involved in compartmentalization, storage, and remobilization of amino acids in plants is still unclear. Two putative cationic amino acid transporters (CAT2, CAT4) were identified in proteomic studies in *Arabidopsis* as incorporated in the tonoplast (Carter et al., 2004; Jaquinod et al., 2007), while the latter study also identified CAT8 and CAT9 in the vacuolar proteome. Green fluorescent protein (GFP)-fusions of CAT2, CAT4, and CAT8 localized at least in part to the tonoplast, confirming these studies (Su et al., 2004; Yang et al., 2014a). The small family of CAT genes comprises 9 genes in *Arabidopsis*, of which the gene products of CAT1, CAT5, and CAT6 were localized at the plasma membrane (Su et al., 2004; Hammes et al., 2006; Yang et al., 2014b) and CAT3 was localized to the endoplasmic reticulum (Yang et al., 2014a). Although amino acids are not *de novo* synthesized in the vacuole, peptides are degraded in the vacuolar lumen and may lead to an increased pool of amino acids in that compartment.

In this study, we identified a peculiar intracellular, mostly vesicular, but minor tonoplast localization of AtCAT9. By using *knock-out* and over-expressor lines of *CAT9*, the gene was identified to be involved in regulating total leaf amino acid concentrations, especially under restricted nitrogen supply. Interestingly, the over-expression of the gene delayed leaf death and improved plant survival after nitrogen starvation. The data suggest that the amino acid homeostasis is critically influenced by intracellular vesicles that contain CAT9. Vesicular/vacuolar nitrogen pools may be a target for improving nitrogen efficiency of crops by manipulating intracellular amino acid storage and mobilization.

## Materials and Methods

### Plant Growth (Soil)

*Arabidopsis thaliana* (*Wassilewskija*, *WS*) wild type and mutant plants were first grown in the greenhouse in nutrient rich garden soil to generate seeds. For further plant culture in soil, we chose a calcareous loess sub soil of a Luvisol with low organic matter content (Tesfamariam et al., 2009). The experimental design was a fully randomized block design with five replicates. The plot size was 6 cm (height) × 5 cm (diameter) and contained 200 g of soil. It was fertilized with 150 mg/kg K_2_SO_4_, 80 mg/kg Ca(H_2_PO_4_)_2_, 50 mg/kg MgSO_4_, and 100 mg/kg (control), or with 10 mg/kg (low N) NH_4_NO_3_. Plants were harvested after 9 weeks and root, shoot, stem, and flower dry weights and total N concentrations were measured.

### Plant Growth (Soil Free)

*Wassilewskija* seeds were vernalized for 48 h at 4^∘^C. For growth in climate chambers on axenic phytoagar plates with modified Hoagland media or in nutrient solution culture with Hoagland media they were surface-sterilized (Yang et al., 2014a). The modified Hoagland media contained all essential nutrients and nitrogen at variable amounts. Plants were maintained at 8 h light, 16 h dark at 22^∘^C and relative humidity 60%. For agar plates, 1 mM NH_4_NO_3_ was used as the sole nitrogen source. For the experiments with nutrient solutions, plants were grown for 5 weeks in 4 l pots. Seeds were positioned on top of a 100 μl drop of full nutrient agar, which was placed in the center of a lid containing a hole that was sufficiently large that the root grew through it after germination. The nutrient solution was either without nitrogen (only with the starter dosage in the agar drop) or with 1 mM NH_4_NO_3_. After the first 2 weeks, the nutrient solution was exchanged every 3 days. In some experiments the plants cultivated for 6 weeks with nitrogen were then transferred to pots without nitrogen for 2–4 further weeks.

### DNA Cloning

The full *cDNA* sequence of *CAT9* (*At1g05940*) that missed the STOP codon was cloned via BamHI and SmaI using the pCRblunt kit (Invitrogen) and inserted 5′ in frame of the *GFP* sequence in a plant binary vector (*pUTkanGFP*) that uses the *ubiquitin10* promoter and kanamycin resistance for plant expression. The fragment containing the entire *CAT9–GFP* fusion sequence was excised with BamHI and PstI and inserted into the into the pDR196 yeast expression vector (Su et al., 2004). Furthermore, a 630 base pair promoter fragment of *CAT9* was isolated and cloned via BamHI in front of the start ATG of the *uidA* [glucuronidase (*GUS*)] gene in the binary expression vector pTkan. Fragment exchange between these vectors allowed the generation of a plasmid containing the endogenous *CAT9* promoter driving the *CAT9* gene. All these constructs were sequenced to exclude PCR errors and transgenic homozygous plants were generated and analyzed. The constructs were used for expression and localization analyses after transformation by *Agrobacterium tumefaciens* (GV3101).

### Functional Expression in Yeast

A yeast mutant with minimal plasma membrane amino acid transport was used for expression of *CAT9–GFP*, as previously described (Fischer et al., 2002).

### Plant Transformation and Analysis

*Arabidopsis* plants were transformed using *Agrobacterium tumefaciens* strain GV3101 with the floral dipping method (Clough and Bent, 1998). Seeds were collected and germinated on modified Hoagland medium containing 50 μg/ml of kanamycin. Transformants were identified for the resistance to kanamycin and GFP fluorescence and selected for further analyses.

### Loss-of-Function and Mutant Isolation and Analysis

The *T-DNA* insertion allele *cat9-1* (FLAG_531A02) was obtained from the Versailles collection in the *WS* background. Homozygous lines were isolated by self-pollination and confirmed by reverse transcriptase PCR. PCR was performed at an annealing temperature of 55^∘^C with 35 cycles. Primer sequences were: (5′–3′): *ACT2-Fw: GTGGGAATGGAAGCTGC TGG, ACT2-Rv: GACCTGCCTCATCATACTCGG, CAT9-Fw: ATGGGAGGCCACGAAGGTTTCAGCAACC, CAT9-Rv: GCTA CATCAATTTCAAAAGCACCGGCA.*

Lines ectopically over-expressing *CAT9* using a ubiquitin promoter were isolated (*pUbq10::CAT9–GFP)* in the WS background. Two lines, which segregated in a Mendelian 3:1 ratio on kanamycin were arbitrarily chosen for further analyses. The homozygous line analyzed for amino acids had 10-fold higher gene expression levels than wild type, as confirmed by reverse transcription PCR. For all plant growth comparisons, the *WS* background was chosen. A cross was made with plants expressing a red fluorescence protein marker and the F1 generation of the cross was analyzed.

Histochemical assays for β-GUS activity (for promoter analysis) were performed using a GUS staining solution with 100 mM sodium phosphate (pH 7), 10 mM EDTA, 3 mM K_4_(Fe(CN)_6_), 0.5 mM K_3_(Fe(CN)_6_), 0.1% (v/v) Triton X-100, 2 mM 5-bromo-4-chloro-3-indolyl-β-D-glucuronic acid (X-Gluc, Roth, Germany) for 12–24 h at 37 C in dark. Vacuum was applied for 30 min. to facilitate substrate infiltration. Tissues were cleared in 70% EtOH.

### N Content and SPAD Analysis

The concentration of total nitrogen was determined using an elemental analyzer (HEKAtech, Wegberg, Germany). The chlorophyll fluorescence was measured with a SPAD meter (Minolta).

### Transient Expression in Suspension Cells

The plasmid encoding the fusion protein CAT9–GFP and marker plasmids were transiently brought by chemical shock into protoplasts from an *Arabidopsis* suspension cell culture. Two days after the PEG-mediated plasmid transformation, protoplasts were analyzed by confocal microscopy (Su et al., 2004).

### Confocal Laser Scanning Microscopy (CLSM) Analysis

The transformed plants were analyzed by confocal microscopy (Leica DMRE microscope equipped with a confocal head TCS SP2; Leica, Wetzlar, Germany). Confocal images were obtained with Leica Confocal software and a 63× water- immersion objective. The excitation wavelength was 488 nm/568 nm; emission was detected for GFP between 500 and 530 nm, for mRFP between 620 and 680 nm. Image processing was done with Adobe Photoshop (Adobe Systems Software Ireland Ltd.).

### Amino Acid Analyses

Fresh leaf material was frozen in liquid nitrogen and amino acids were extracted as described (Yang et al., 2014b).

### Electron Microscopy

Immunogold-labeling was performed on ultrathin (80–100 nm) thawed Tokuyasu cryosections of formaldehyde (8%, 2 h) fixed and sucrose-infiltrated (2.1 M) root tips using rabbit anti-GFP serum [1:250, 60 min; (Abcam)] and silver-enhanced (HQ Silver, 8 min; Nanoprobes) goat (Fab’) anti-rabbit IgG coupled to Nanogold (1:50; No 2004, Nanoprobes). Staining with uranyl acetate and final embedding in uranyl acetate/methylcellulose (Sigma) was done as previously described (von der Fecht-Bartenbach et al., 2007).

### Statistical Analysis

Error bars show the SD throughout. The data were analyzed by ANOVA with LSD test using the SAS program (Statistical Analysis System, Version 9.4). Statistically significant differences are indicated with different characters. Small letters indicate statistically different values at *p* < 0.05 and capital letters at *p* < 0.01.

## Results

### Broad Expression Pattern and Intracellular Localization

**FIGURE 1:**
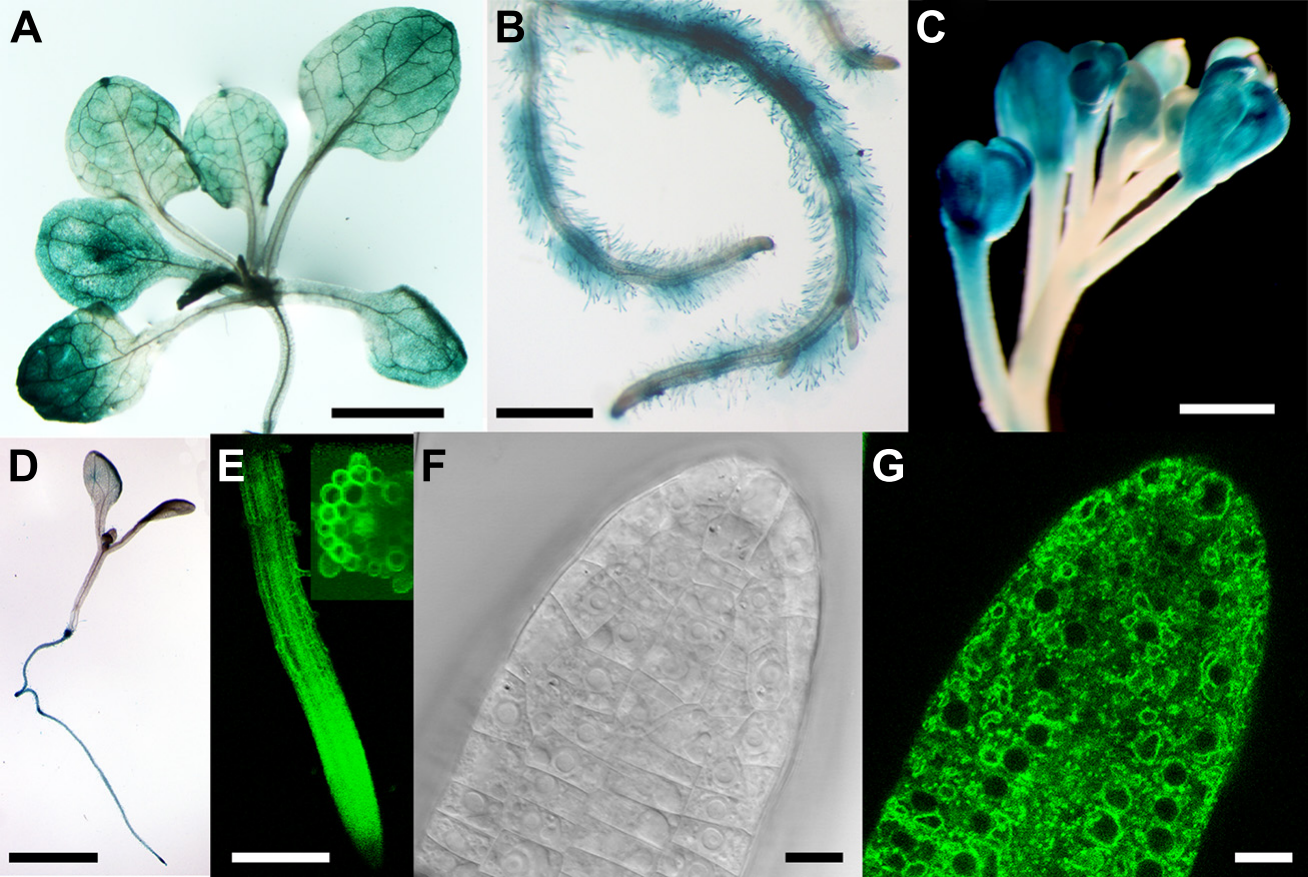
**Tissue expression pattern of *CATIONIC AMINO ACID TRANSPORTER* (*CAT9*)**. Histochemical glucuronidase (GUS) analysis from the shoots of a 25-days-old seedling expressing the p*CAT9::GUS* construct **(A)** and roots **(B)**. Staining of flowers **(C)** and of a 4-days old seedling **(D)**. **(E)** Fluorescence from 10-days old seedling roots expressing *pCAT9::CAT9–GFP;* inset: 40-stacks-sectioned fluorescence). **(F,G)** Bright field and fluorescence image of a *pCAT9::CAT9–GFP* expressing root tip. Scaling bars: **(A**,**B**,**D)**: 0, 5 cm, **(C,E)**: 250 μm, **(F,G)**: 20 μm.

The expression pattern of* CAT9* was first analyzed using the β-GUS reporter, driven by the endogenous upstream promoter. The blue color, indicating the promoter activity, showed a broad expression in the shoot and root throughout development, including root hairs (**Figures 1A,B**). The promoter activity was also found in the reproductive organs, the flowers (**Figure 1C**). An even broader expression pattern is supported by a large number of microarrays, with little variation in the expression level at different developmental stages and within different organs (Schmid et al., 2005). In very young seedlings, the promoter activity was primarily found in the root, but not dominant in the shoot (**Figure 1D**). When expressed as a translational fusion with GFP under the same endogenous promoter, the fluorescence of the plants again supported the broad expression pattern. In the root of 10 days old seedlings, the promoter was active throughout the root tissues (**Figure 1E**). When analyzed at higher resolution, the fluorescence was found mostly in a punctuate fashion, but some labeling was identified also at vacuolar, intracellular structures (**Figures 1F,G**).

### Primary Localization in Vesicles, Including the *Trans*-Golgi Network

**FIGURE 2:**
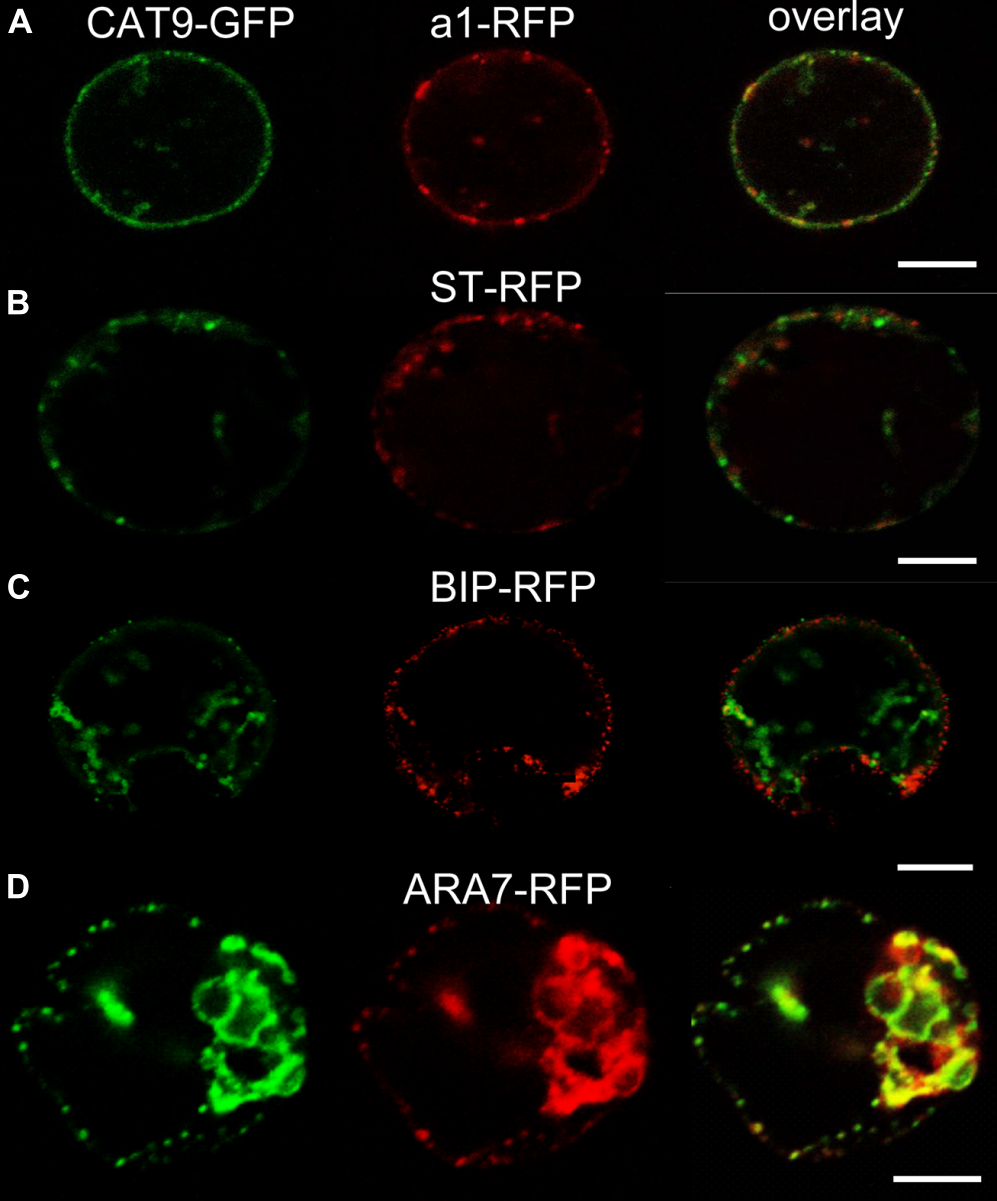
**Sub-cellular co-localization of *pCAT9::*CAT9–GFP (green, left) with red fluorescent protein (mRFP) -labeled markers (red, middle) and overlap (right) in transiently expressing protoplasts. **(A)**** *Trans*-Golgi network (TGN) marker V-ATPase subunit a1-RFP, **(B)** Golgi marker sialyl transferase (ST-RFP), **(C)** endoplasmic reticulum marker BIP-RFP, **(D)** endosomal marker ARA7-RFP. Scaling bars: 10 μm.

The identity of the punctuate compartment labeled by CAT9–GFP was identified by transient co-expression of red fluorescent protein (mRFP) labeled marker constructs for different internal compartments in protoplasts. The punctuate pattern was also seen in protoplasts transiently transformed with the translational *CAT9–GFP* construct (**Figure 2**). A significant, but not complete signal overlap with punctuate fluorescent structures that were labeled by the mRFP-tagged VHA-a1 subunit of the V-ATPase was observed. This construct serves as a *trans-*Golgi network (TGN) marker (Dettmer et al., 2006). By contrast, only a very limited signal overlap was found with the rat sialyl transferase (ST-mRFP) marker that labels the *trans*-Golgi cisternae and the *TGN* in plants (Saint-Jore et al., 2002). Likewise, co-expression with the mRFP-labeled endoplasmic reticulum marker BIP (Jin et al., 2001) did not show overlap (**Figure 2C**). Furthermore, significant co-staining was also detected with mRFP-tagged ARA7 (AtRabF2b, a Rab-type GTPase), which localizes to the limiting membrane of multivesicular bodies (MVBs) and has minor overlap with the TGN (Kotzer et al., 2004; Ueda et al., 2004; Scheuring et al., 2011).

**FIGURE 3:**
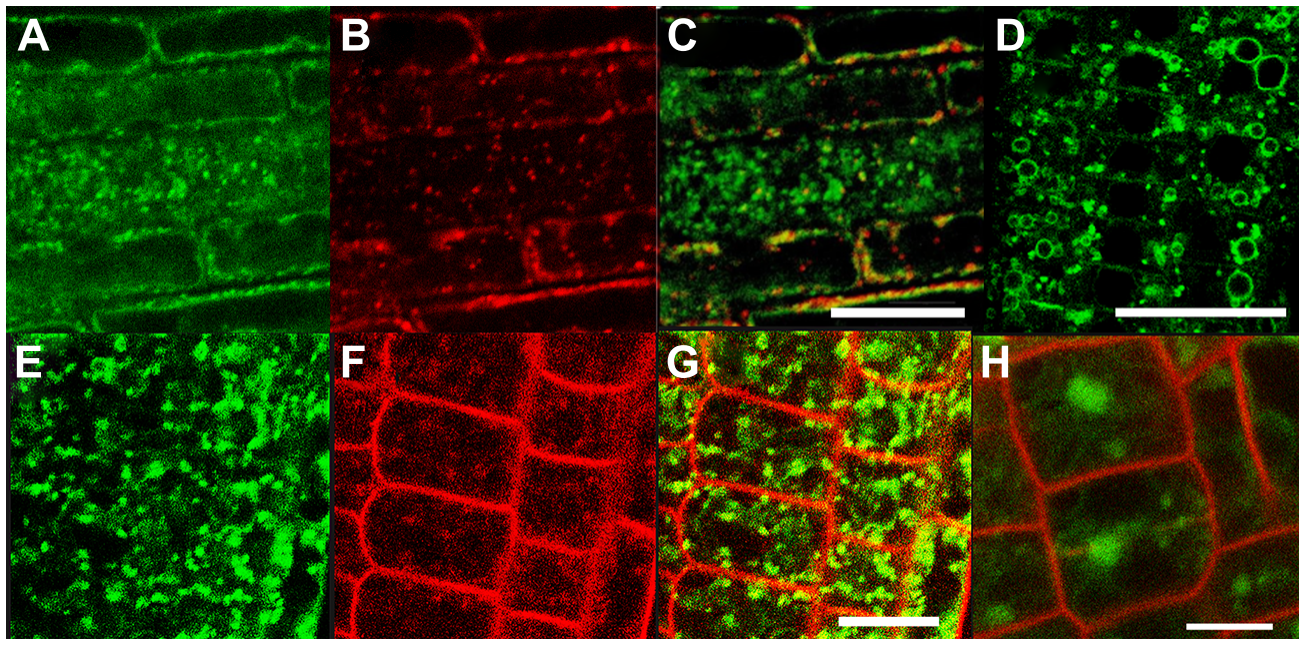
**The compartment labeled by CAT9–GFP in roots**. Punctuate fluorescence labeling of *pCAT9::*CAT9–GFP (**A**, green) and ARA7-mRFP (**B**, red) and partial overlap **(C)**. CAT9–GFP fluorescence after concanamycin A treatment (100 nM for 30 min, **D**). Punctuate *pUbq10::*CAT9–GFP fluorescence **(E)**, FM4-64 (**F**, 15 min. after addition, red), and partial overlap **(G)**. Aggregation of the green CAT9–GFP fluorescence into one or two spots per cell after brefeldin A treatment for 2 h (**H**, red: propidium iodide). Scaling bars: **(A–H)**: 20 μm.

**FIGURE 4:**
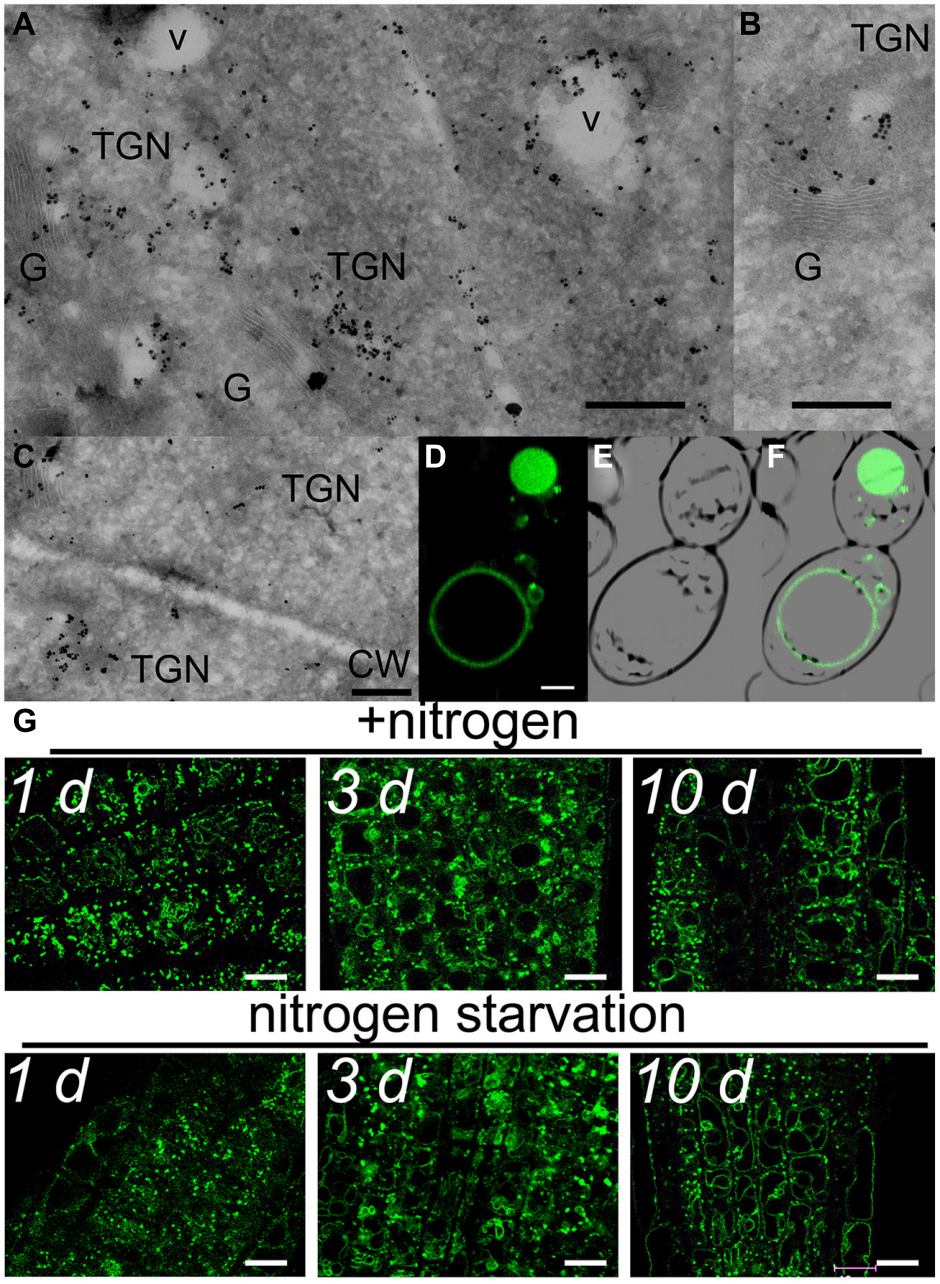
**Subcellular localization of CAT9–GFP**. Transmission electron microscopic images of ultrathin root cryosections of immunogold-labeled *pUbq10::*CAT9–GFP **(A,B,C)**. v, vesicular structure; TGN, *trans*-Golgi network; G, Golgi; CW, cell wall with adjacent plasma membranes. The construct was expressed from its endogenous promoter Scaling bars: 500 nm. **(D)** Fluorescence of heterologously expressed *CAT9–GFP* in yeast, **(E)** bright field image, **(F)** overlay. Scaling bar: 2 μm. **(G)** Fluorescence pattern of *pCAT9::*CAT9–GFP in roots a few mm behind the root tip in the presence of nitrogen (upper row) or after transfer to plates lacking nitrogen for 1, 3, or 10 days (lower row). Scaling bars: 10 μm. The complemented line (cl) in the *cat9-1* background was used.

This partial overlap of CAT9–GFP (driven by its native promoter) with ARA7-mRFP was confirmed when both constructs were stably co-expressed in the same roots (**Figures 3A–C**). The V-ATPase inhibitor concanamycin A, which significantly reduces the number of MVBs and causes TGN and MVB markers to overlap (Scheuring et al., 2011), moderately reduced the small punctuate structures and caused CAT9–GFP to be localized to larger vesicular structures (**Figure 3D**). The mostly punctuate fluorescence of CAT9–GFP was also seen when the gene fusion was over-expressed, driven by the *ubiquitin10* promoter (**Figures 3E–H**). The CAT9–GFP compartments also partially overlapped with intracellular vesicles that appeared after adding the membrane dye FM4-64 (2 μM) to the cells (**Figures 3E–G**). This lipid marker primarily stains the cell plasma membrane, but early endocytotic *TGN* vesicles are also stained after a few minutes after dye addition. This indicates that the membrane dye is incorporated rapidly via endocytosis into vesicular structures (Dettmer et al., 2006). Furthermore, part of the CAT9-compartments were sensitive to the vesicle transport inhibitor Brefeldin A (BFA, 50 μg/ml, 2 h, **Figure 3H**), which induced reversible aggregation of the fluorescence in one or two spots per cell. As MVBs are not part of BFA compartments, but the TGN is, this probably reflects the major localization in the TGN (Scheuring et al., 2011). This sub-cellular localization was confirmed with transmission electron microscopy on immunogold-labeled ultrathin cryosections of roots. Using an anti-GFP antibody, a consistent labeling with gold particles close to tubulovesicular structures was seen at the trans site of the Golgi stacks in the *TGN* (**Figures 4A,B**). Furthermore, intense labeling of internal small vesicular structures (v), but not of plasma membranes (**Figure 4C**), was also observed. The results are consistent with a preferential localization of CAT9–GFP in vesicles that are involved in vacuolar trafficking, including the highly dynamic *TGN* vesicles.

Whether cell age or the nitrogen status influenced the fluorescent pattern, was tested in 10 days-old plants that were transferred from complete medium to nutrient plates lacking nitrogen. A weak tendency for more pronounced localization in tonoplast membranes in older cells was detected, but the typical punctuate CAT9–GFP fluorescence pattern with minor localization at the tonoplast did not differ even after 10 days of nitrogen starvation (**Figure 4G**).

### Putative Function as Intracellular Amino Acid Transporter

When heterologously expressed in yeast, the fluorescence was detected in small punctuate structures and at the vacuolar membranes (**Figures 4D–F**). Despite that CAT9 was incorporated in intracellular membranes, we attempted to measure its transport function in a yeast mutant that was defective in amino acid uptake at the plasma membrane. The chosen yeast strain lacks eight endogenous amino acid transporters, but is competent to synthesize all amino acids (Fischer et al., 2002). However, the growth of *CAT9–GFP*-transformed yeast cells was not different on several selective media with specific amino acids as sole nitrogen source compared with empty plasmid transformed controls (*data not shown*), which may be easily explained by its localization in internal membranes.

### Identification of a *Loss-of-Function* Mutant* cat9-1* and Over-Expressors

**FIGURE 5:**
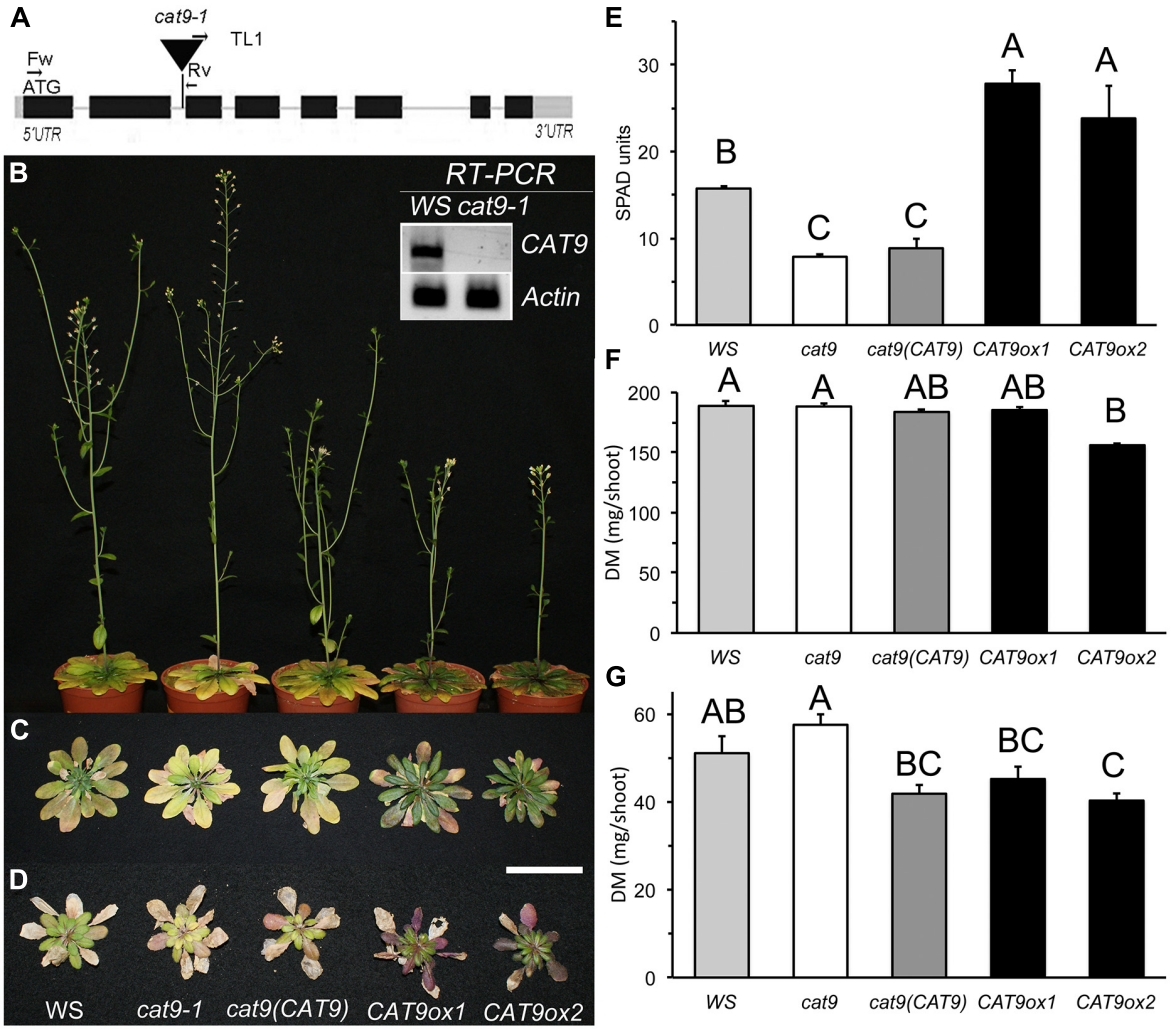
**Identification of *knock-out* and over-expression lines for *CAT9* and their growth characteristics on soil. **(A)**** Schematic view of *CAT9* genomic structure (2823 bp). Black squares: exons, gray squares: untranslated regions. The *T-DNA* insertion was localized in the second intron. Inset: total loss of CAT9 transcript in the homozygous *cat9-1* mutant. *Actin2* served as a control. **(B)** Nine-week old plants on fertilized soil at flowering, from left to right: *Wassilewskija (WS*) wild type, *cat9-1*, *cl cat9 (CAT9*) and two over-expressor lines *CAT9ox1* and *CAT9ox2*. **(C)** Rosettes from the same lines and from plants grown on low nitrogen **(D)**. Scaling bar: 5 cm. **(E)** Chlorophyll fluorescence in SPAD values (for Soil Plant Analysis Development). **(F)** Dry shoot biomass (DM) of N-fertilized soil-grown plants and **(G)** DM of low-N soil-grown plants. Statistically different values (ANOVA, *n* = 5) are given with different capital letters at *p* < 0.01 and with different lower case letters at *p* < 0.05.

A *loss-of-function* allele was identified in the Versailles collection of *T-DNA* insertion lines in the *WS* background. Homozygous plants for the insertion were isolated after selfing and selecting from a heterozygous parent. The position of the *T-DNA* insertion in the second intron is schematically shown in **Figure 5A**. Homozygous plants for the *T-DNA* insertion did not contain residual *CAT9* transcript (**Figure 5A**, insert). This suggests that in *cat9-1* the entire *CAT9* expression is lost and the mutant represents a *loss-of-function* mutant. Homozygous *cat9-1* plants lacking *CAT9* expression were then transformed with a plasmid containing the entire *CAT9 cDNA* sequence, translationally fused to the *GFP* sequence at the C-terminus, driven by the endogenous promoter [*cat9-1*(*pCAT9::CAT9–GFP*) *also referred as complemented line, cl*]. Of the homozygous transgenic plants finally obtained, one line with ∼1.4-fold *CAT9* expression line was arbitrarily chosen. In addition, homozygous lines of the *WS* wild type transformed with a plasmid containing the *CAT9–GFP cDNA* sequence driven by the strong ubiquitous *UBIQUITIN10* promoter (*pUbq10*::*CAT9–GFP)* were generated. Plant lines with segregation close to a 3:1 ratio on kanamycin were chosen.

When germinated on synthetic agar medium containing 1 mM NH_4_NO_3_, as sole nitrogen source, no growth differences between mutant and *wild type* were observed. The mutant lines [*knock-out*, cl and over-expressor lines] were then planted on a fertilized “control” sandy loam soil. At mature growth stages, these plants showed visual leaf symptoms, which differed in the mutant and in the over-expressor lines (**Figure 5B**). A slightly later development of the inflorescence in the over-expressor lines is visible by the smaller stem in the representative plants shown. While the leaves of *cat9-1* appeared slightly less green and more yellow after 9 weeks in short day conditions, the two over-expressor lines were darker green and senescence appeared delayed (**Figure 5C**). The chlorophyll content (measured as fluorescence SPAD values) in the *knock-out* lines was slightly lower than in the wild type. By contrast, the SPAD values were much higher in the over-expressors (**Figure 5E**). The shoot biomass did not differ between the wild type, *knock-out*, and *cl*. Furthermore, the biomass among the two over-expressor lines was not significantly different, but slightly reduced compared to the other lines (**Figure 5F**).

When grown with 10-fold less nitrogen fertilizer addition (low N) in the same soil, the shoot growth was strongly inhibited, but the visual differences of the shoot resembled those at higher nitrogen availability (**Figure 5D**). The shoot biomass of the low N-grown plants was higher in the *knock-out* compared to plants with elevated *CAT9* expression (**Figure 5G**).

### Growth with a Low Starting Dosage of N and/or Low Continuous N-Supply in Nutrient Solution

**FIGURE 6:**
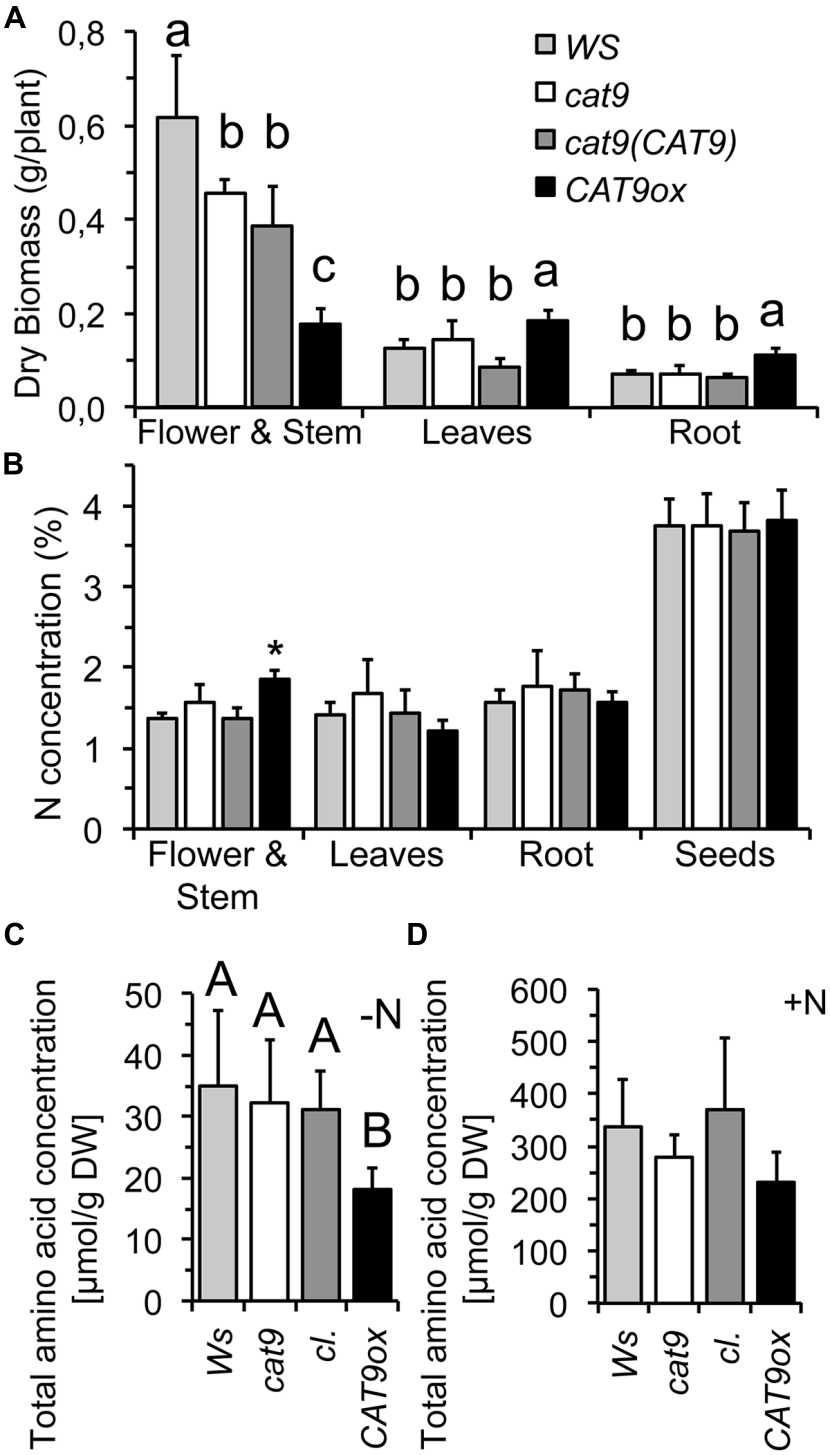
**Biomass, nitrogen, and amino acid concentrations of wild type and mutants in nutrient solution hydroponics**. **(A)** Organ dry biomass of *WS* wild type (light gray bar), *cat9-1* (white bar), *cl* (dark gray bar) and *CAT9ox1* (black bar) after 5 weeks in nutrient solution. **(B)** Nitrogen concentration in different tissues. **(C,D)** Total free amino acid concentration in leaves grown only with a starting dosage of N **(C)** and in leaves from plants grown with 1 mM NH_4_NO_3_ for 5 weeks **(D)**. Statistically different values (ANOVA, *n* = 5) are given with different capital letters or (^∗^) at *p* < 0.01 and with different lower case letters at *p* < 0.05.

As the nitrogen availability generally affects intracellular soluble amino acid levels, the wild type and mutants were compared at different nitrogen level in nutrient solutions. The plants were initially grown with a constant dosage of 1 mM NH_4_NO_3_ in nutrient solutions for 5 weeks in short day conditions. The *CAT9ox1* over-expressor line was chosen for further experiments, which had about 10-fold higher expression of *CAT9*, when analyzed by *RT-PCR.* Compared to the wild type, *cat9-1*, and the cl, the development of the over-expressor was again delayed and resulted in smaller stem and inflorescence. However, this was associated with an increase in leaf and root biomass (**Figure 6A**). The nitrogen concentration in the different tissues was low, reflecting the limited nitrogen supply (1 mM NH_4_NO_3_) and did not differ among the lines, except for the young stems of the over-expressor (**Figure 6B**), which is likely explained by the different (later) developmental stage of the stems in the over-expressor mutant.

In a parallel experiment, plants were initially supplied with a small amount “starting dosage” of N, but were then grown without any further nitrogen, to test whether this initial N supply might be differently distributed in the mutants. However, all plants developed a similar pale green chlorotic appearance and the very little biomass generated by the roots and the shoots of these seedlings did not statistically differ. Interestingly, however, a very low total soluble amino acid concentration was measured in the over-expressor plants, potentially indicating a different amino acid homeostasis (**Figure 6C**). The total soluble amino acid concentrations were also measured in the plants from the other experiment with the nutrient solution containing 1 mM NH_4_NO_3_, but these had similar and not statistically different total free amino acid concentrations in the leaves (**Figure 6D**). A not significant, but consistent trend with lower amino acid pools was, however, also observed in the over-expressor under these conditions.

### Alteration of Leaf Amino Acid Levels by *CAT9* and N-Depletion

**FIGURE 7:**
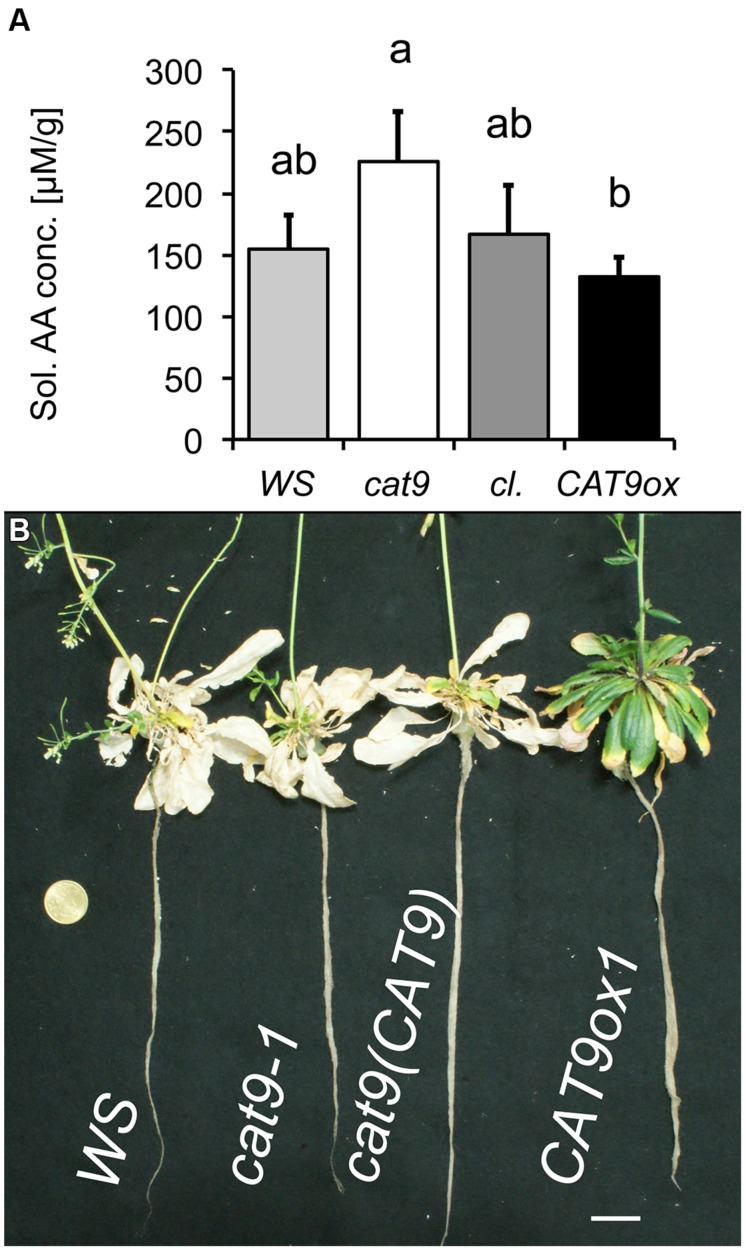
**Nitrogen starvation phenotype of mutant lines. **(A)**** Total soluble amino acid concentration in leaves of plants initially grown for 6 weeks with 1 mM NH_4_NO_3_ and then N-starved for 2 weeks. Bar colors: *WS* wild type (light gray), *cat9-1* (white), *cl* (dark gray), and *CAT9ox1* (black). **(B)** Visual phenotype of mutant plants from the above experiment, but starved for 2 further weeks of nitrogen (4 week N-starvation). Scaling bar: 2 cm. Statistically different means (*n* = 5) at *p* < 0.05 are given with different letters.

Plants were then again grown in nutrient solution with 1 mM NH_4_NO_3_, but after 6 weeks of growth with N supply, these plants were transferred to a solution without nitrogen. Two weeks later, the samples were harvested and soluble amino acids were extracted from the leaves. The amount of soluble amino acids had decreased compared to the situation with N (**Figure 6D**), but higher amino acid levels were detected in the *loss-of-function* mutant, compared to the over-expressor, while those of the wild type and cl were intermediate (**Figure 7A**). Interestingly, *CAT9ox1* plants appeared to suppress leaf death and after 2 additional weeks of N-starvation, only the leaves of *CAT9ox1* mutants were partially green (but covered with necrotic edges), while those of the wild type and other genotypes were dry and dead (**Figure 7B**).

**FIGURE 8:**
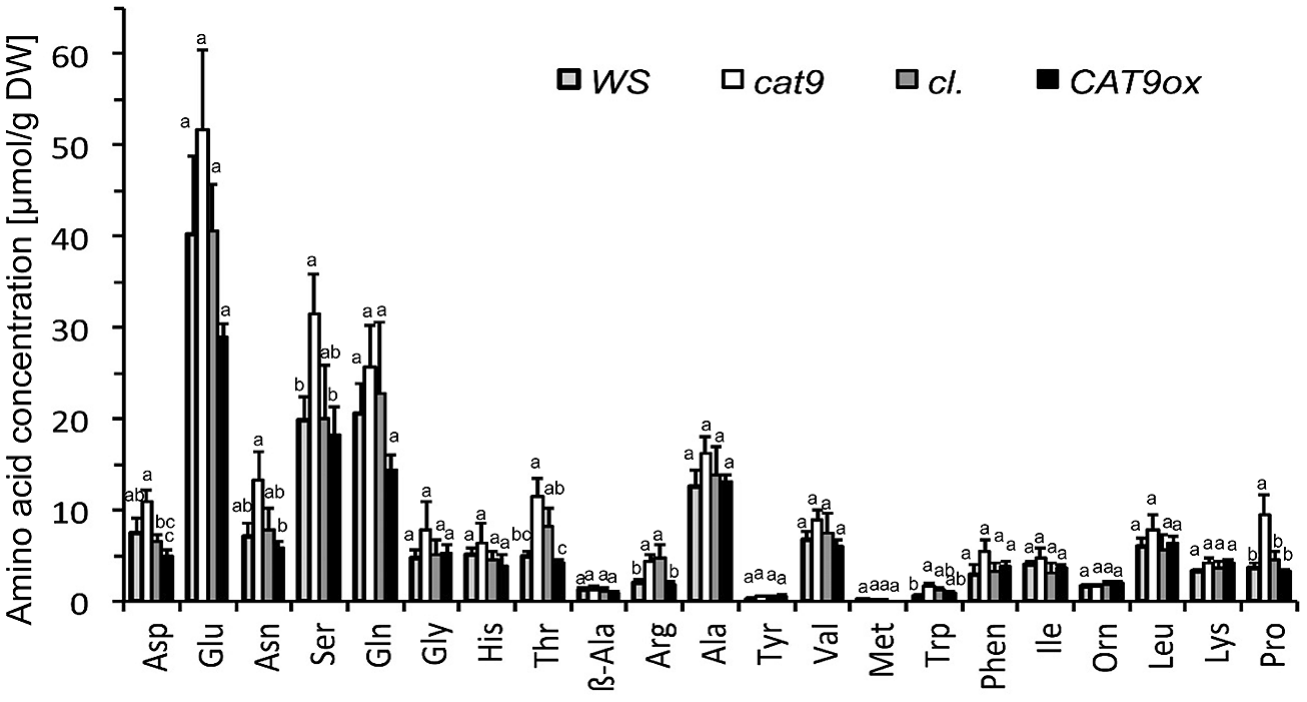
**Amino acid profile of leaves after 2 weeks of nitrogen starvation**. Individual amino acids in μmol per dry weight (*n* = 4). Three letter abbreviation of amino acids, the Ile data points overlap with OH-Lys. Note the significant differences in some amino acids (*p* < 0.05, ANOVA). Bar colors: *WS* wild type (light gray), *cat9-1* (white), *cl* (dark gray), and *CAT9ox1* (black).

A consistent trend for different amounts in the total soluble amino acid pools after 2 weeks of nitrogen starvation was observed in several individual amino acids. Significant differences were measured for Asp, Asn, Ser, Thr, Arg, and the cyclic amino acid Pro between the *loss-of-function* mutant and the over-expressor (**Figure 8**), which is in line with the total elevation of the soluble amino acids in the *knock-out* mutant and a decrease of these amino acids in the over-expressor after N starvation. Some of these elevated amino acids serve as long-distance “transport amino acids,” but at least at constant supply of 1 mM NH_4_NO_3_, the root (and seed) were not differently supplied with nitrogen (**Figure 6B**).

## Discussion

### Localization of *CAT9* and Impact on Plant Growth

Several putative amino acid transporters of the family of *CATs* are localized to intracellular compartments, including CAT2 and CAT4, which localize to the tonoplast (Yang et al., 2014a). CAT2 may have a function in transport or re-mobilization of amino acids across the tonoplast, as total soluble leaf amino acid concentrations are altered in the *cat2* mutant (Yang et al., 2014a). In contrast to all other so far characterized putative plant amino acid transporters, CAT9 localized primarily to intracellular vesicles, including those of the *TGN* and “pre”-vacuolar structures, which appear to have a role in vacuolar trafficking (**Figures 2–4**). A minor localization to vacuolar membranes was also encountered, with the weak trend that this fraction was increased with aging, explaining why CAT9 had been identified in the vacuolar proteome (Jaquinod et al., 2007). The current data do not rule out that CAT9 might also be localized at autophagosome membranes, which also merge with lytic vacuoles and accumulation of which is also modified by concanamycin A treatment. Autophagy is crucially involved in protein and amino acid remobilization and turnover. The CAT9–GFP localization pattern was not dependent on the expression strength, as it did not differ when the gene was expressed from its endogenous promoter in the *cat9-1* mutant background or was over-expressed in the wild type (**Figure 4**). *CAT9* promoter activity was observed in roots, especially root hairs, shoots and reproductive organs, but the gene expression prediction from microarrays suggests even a more uniform broad expression pattern. It is possible that further downstream or upstream regulatory elements influence the gene expression pattern of functional *CAT9*.

Homozygous *cat9-1* plants were identified that had lost the entire *CAT9* expression (**Figure 5**). These mutants were characterized by minor growth differences and chlorotic leaves, which depended on the nutritional status, as visual symptoms were initially not observed in nutrient-rich garden soil. Reintroduction of the *CAT9–GFP* fusion construct under the endogenous promoter into the mutant only partially complemented the phenotypes, but strong ectopic over-expression using the ubiquitin promoter reversed the phenotypes seen in the *loss-of-function cat9-1* mutant. Over-expression of *CAT9* delayed the formation of stems and inflorescence, but increased leaf and root dry biomass.

### Vesicular Amino Acid Transport and Cellular Homeostasis

Intracellular vesicles might directly participate in intracellular amino acid homeostasis. If so, a *loss-of-function* line and over-expressor should affect the intracellular pools of free amino acids. Alternatively, *CAT9* might be somewhat involved in the trafficking of endosomal vesicles, and *loss-of-function*, or over-expression might delay the development, which then alters plant biomass, bolt height, green leaves, ability to cope with nitrogen starvation, and indirectly might affect free amino acid concentrations.

A distinction between these hypotheses, and whether CAT9 directly or indirectly affects amino acid pools, is not yet possible, as the amino acid concentrations in small cellular vesicles in plants remain unknown for technical reasons and the direct transport function of CAT9 remains unknown. It may be possible that in the future, *in vivo* measurements of metabolite levels in small compartments get available by the use of fluorescent amino acid detectors targeted specifically to unique compartments (Okumoto et al., 2005; Bogner and Ludewig, 2007; Yang et al., 2010). However, the pH-sensitivity of these GFP-derived reporters and problems to specifically address the compartment lumen of choice have, until now, hindered their application.

### Nitrogen Storage, Remobilization, and Cellular Amino Acid Homeostasis

The soluble amino acid concentrations remained higher in *cat9-1* compared to the over-expressor after N starvation, pointing to a (direct or indirect) effect on the cellular amino acid homeostasis and remobilization, which may be most relevant in the leaves. Significant differences in the soluble amino acid leaf pools of leaves occurred in Asp, Asn, Ser, Thr, Arg, and the cyclic amino acid Pro. These amino acids typically vary diurnally, also increase upon nitrogen fertilization and may change with development. That these amino acids represent the substrate profile of CAT9 is considered as unlikely, as the small vesicular luminal compartment is too small in its volume to significantly accumulate (or exclude) large amounts of amino acids. It is more likely that the metabolism of individual amino acid levels is adjusted in the mutants. If so, vesicular pools may have a role in controlling overall soluble amino acid homeostasis. However, it is important to note that a minor fraction of CAT9–GFP was additionally found at the vacuolar membrane, so that we cannot rigorously exclude that the phenotype is related to its function as a vacuolar transporter.

Nitrogen recycling and remobilization are differentially controlled by leaf senescence and development stage (Diaz et al., 2008). Many soluble amino acids, such as Lys, have strong intrinsic inhibitory effects on the plant metabolism, as their biosynthesis pathways are strongly feedback-controlled (Bonner et al., 1992). The selective storage of such inhibitory amino acids in vacuoles or vesicles may be a tool to bypass the metabolic inhibition.

Interestingly, a mutant in the gene nitrogen limitation adaptation (*nla*), which codes for a ubiquitin ligase involved in protein degradation and thus N recovery, showed a somewhat similar phenotype as *cat9-1* (Peng et al., 2007). It will be interesting to investigate whether these genes are part of the same pathway in the adaptation to low N.

The weak phenotype of the *knock-out* of the predominantly vesicular localized amino acid transporter gene *CAT9* appears to suggest that this gene is dispensable under many physiological conditions, but the finding that its over-expression delays development and extends the leaf lifetime under severe N shortage may have practical relevance when transferred to crops. Although little is known about molecular details of intracellular and vesicular amino acid transport and storage in crops, genome wide association mapping of complex metabolic traits has recently implicated a homolog of a vacuolar *CAT* gene in maize amino acid levels (Riedelsheimer et al., 2012).

## Conflict of Interest Statement

The authors declare that the research was conducted in the absence of any commercial or financial relationships that could be construed as a potential conflict of interest.
